# Aβ Oligomer Toxicity-Reducing Therapy for the Prevention of Alzheimer’s Disease: Importance of the Nrf2 and PPARγ Pathways

**DOI:** 10.3390/cells12101386

**Published:** 2023-05-13

**Authors:** Wataru Araki

**Affiliations:** 1 Department of Neurology and Neurological Science, Tokyo Medical and Dental University, Bunkyo-ku, Tokyo 113-8510, Japan; wataruara@yahoo.co.jp; 2 Memory Clinic Ochanomizu, Bunkyo-ku, Tokyo 113-8510, Japan

**Keywords:** Alzheimer’s disease, amyloid beta, neuroinflammation, Nrf2, oligomer, PPARγ, small molecule, toxicity

## Abstract

Recent studies have revealed that soluble amyloid-β oligomers (AβOs) play a pathogenetic role in Alzheimer’s disease (AD). Indeed, AβOs induce neurotoxic and synaptotoxic effects and are also critically involved in neuroinflammation. Oxidative stress appears to be a crucial event underlying these pathological effects of AβOs. From a therapeutic standpoint, new drugs for AD designed to remove AβOs or inhibit the formation of AβOs are currently being developed. However, it is also worth considering strategies for preventing AβO toxicity itself. In particular, small molecules with AβO toxicity-reducing activity have potential as drug candidates. Among such small molecules, those that can enhance Nrf2 and/or PPARγ activity can effectively inhibit AβO toxicity. In this review, I summarize studies on the small molecules that counteract AβO toxicity and are capable of activating Nrf2 and/or PPARγ. I also discuss how these interrelated pathways are involved in the mechanisms by which these small molecules prevent AβO-induced neurotoxicity and neuroinflammation. I propose that AβO toxicity-reducing therapy, designated ATR-T, could be a beneficial, complementary strategy for the prevention and treatment of AD.

## 1. Introduction

Alzheimer’s disease (AD) is considered a critical health problem in an aging society. The neuropathology of AD is characterized by the presence of senile plaques and neurofibrillary tangles. Senile plaques are extracellular deposits principally composed of amyloid-β (Aβ) peptides, and neurofibrillary tangles are intraneuronal aggregations mainly composed of abnormally phosphorylated tau protein. Aβ is generated by two-step proteolytic processing of amyloid precursor protein (APP) by β-secretase, BACE1 (β-site APP cleaving enzyme 1), and γ-secretase complexes, comprising presenilin1 (PS1) or 2 (PS2) and nicastrin, Aph1, and Pen2. Aβ40 and Aβ42 are major Aβ species, with the latter being more aggregable and pathogenic [1,2]. Aβ is cleared from the brain through various mechanisms, including proteolytic degradation, clearance by glial cells, transport across the blood–brain barrier (BBB), perivascular drainage, and clearance through the glymphatic system. Although Aβ production is known to be affected in familial AD, an imbalance between the production and clearance of Aβ appears to be involved in sporadic AD [3].

Recent AD research has established that significant Aβ accumulation has already occurred at the prodromal stage of AD, known as mild cognitive impairment (MCI) due to AD, followed by the spreading of abnormal tau protein to parietotemporal areas in the cerebral cortices [4,5]. Further, recent evidence indicates a significant contribution of neuroinflammation to AD pathogenesis. For example, genetic studies have identified gene variants of the microglial receptor TREM2 (triggering receptor expressed on myeloid cells 2) as risk factors for developing AD [6,7]. The link between Aβ and tau has been a matter of intense investigation. One important and plausible theory is that soluble assemblies of Aβ called Aβ oligomers (AβOs) are potent toxic species that not only induce tau abnormalities but also promote synaptic disturbances and neuroinflammation [8,9,10,11]. It has been accepted that AβOs exert much greater toxicity than Aβfibrils [12]. AβOs exist in AD brain tissues [8,9], are tightly linked to Aβ plaque pathology in AD brains [13], and may be sequestered into senile plaques [14]. Because the Aβoligomer hypothesis offers a reasonable explanation for the pathophysiological alterations in AD brains, it has led to the design of new therapeutic approaches that target AβOs. Such approaches include eliminating AβOs through immunological methods and inhibiting the formation of AβOs by modulating Aβ assembly [9,15,16]. In addition to these approaches, reducing the intrinsic toxicity of AβOs using certain small molecules is also a potential strategy. Indeed, a number of recent preclinical studies have suggested the viability of this latter approach [17]. Since Aβ accumulation has reached a substantial level by the prodromal stage of AD, it is particularly important to start therapeutic intervention as early as possible to prevent the clinical progression to AD dementia.

In a previous review, I discussed potential mechanisms underlying the action of AβO neurotoxicity-reducing small molecules [17]. Notably, almost all of these small molecules possess antioxidative properties, and most can stimulate the activity of Nrf2 (nuclear factor erythroid 2-related factor 2) [18], which is essential in antioxidative defense mechanisms. Furthermore, some of these molecules are capable of activating peroxisome proliferator-activated receptor-gamma (PPARγ), which has a wide spectrum of functions that include antioxidative defense [19,20]. Indeed, these two pathways are interrelated, as described below. In the present mini-review, I first briefly discuss the significance of AβOs in AD pathogenesis and the characteristics of the small molecules that can reduce AβO toxicity. Then, I specifically review those small molecules that can activate Nrf2 and/or PPARγ and discuss their characteristic properties as well as their potential as drug candidates for the prevention of AD. 

## 2. AβOs Play a Key Role in AD Pathogenesis 

It is well recognized that AβOs play significant roles in the pathogenetic mechanisms of AD, reflecting their ability to elicit neurotoxicity, synaptotoxicity, and neuroinflammation and the fact that these effects can account for the neuropathological features of AD [8,9]. AβOs are mixtures of heterogeneous species, ranging from small to large; however, which species are the most toxic remains to be elucidated [9,11]. Despite these uncertainties, targeting AβOs is a reasonable strategy for developing therapeutic drugs for AD, as mentioned above.

The first point to emphasize regarding mechanistic aspects of AβO toxicity is that the neurotoxicity of AβOs involves synaptic degeneration and tau abnormalities. Although the mechanisms underlying these effects have not yet been clarified, the most plausible mechanism is that AβOs bind cell-surface receptors, such as NMDA (N-methyl-D-aspartate) receptors and PrP^c^ (cellular prion protein), inducing various downstream pathological events, including oxidative stress, Ca^2+^ dyshomeostasis, mitochondrial dysfunction, apoptosis, synaptic disruption, and tau abnormalities [9,21,22]. Among these events, oxidative stress appears to have a central role, given that oxidative stress is a pathological feature of the earlier stages of AD, including MCI due to AD [23,24]. The mechanisms by which AβOs induce oxidative stress remain to be elucidated, but many studies have shown that exposure to AβOs causes the production of reactive oxygen species (ROS), most likely as a result of mitochondrial dysfunction [25]. AβOs induce Ca^2+^ dyshomeostasis not only in the cytosol but also in mitochondria, and an increase in Ca^2+^ influx into mitochondria via the mitochondrial Ca^2+^ unipolar complex may lead to mitochondrial dysfunction [26,27]. AβOs also disrupt mitochondrial dynamics (e.g., fusion and fission) and energy metabolism [25]. Furthermore, cytochrome C release from mitochondria promotes apoptosis. It is also well established that AβOs induce tau abnormalities that can be at least partly explained by activation of tau kinases—a process in which oxidative stress plays a significant role [28]. It is additionally noteworthy that AβOs induce neuronal insulin resistance, which may underlie the impaired insulin signaling in AD [9,29,30].

The second point worth stressing is that AβOs trigger neuroinflammation through the activation of glial cells, especially microglia. The binding of AβOs to microglia, which appears to be mediated by the receptors CD36, TLR4 (Toll-like receptor 4), and TLR6, results in their activation and consequent production of proinflammatory cytokines and chemokines [6,7,31,32]. Oxidative stress also appears to mediate the proinflammatory action of AβOs, given that AβOs induce ROS in microglia through activation of NADPH oxidase (NOX) and promote mitochondrial damage, as well as the fact that ROS can activate caspase 1 and NLRP3 (NOD-like receptor protein 3), the latter of which forms an important component of the innate immune response to pathogens called the inflammasome [33]. Heneka and co-workers also demonstrated that AβOs activate NLRP3 inflammasomes in microglia [34]. In addition, a recent study found that conditioned medium from AβO-stimulated microglia elicits necroptosis in neurons, further supporting the important pathological role of microglia in AD [35].

Notably, recent studies have revealed that AβOs are closely associated with microglia through TREM2, a cell-surface receptor on microglia that engages in innate immune responses, including phagocytosis, chemotaxis, and transcriptional changes [6,7]. Recent studies have shown that TREM2 is a receptor of AβOs, demonstrating that binding of AβOs to TREM2 activates TREM2-dependent signaling pathways and modulates microglial responses such as migration and phagocytosis. Interestingly, AD-associated mutations in TREM2 reduce TREM2 binding to AβOs [36,37]. The binding of AβOs to microglia also induces the shedding of the TREM2 ectodomain. This leads to the production of soluble TREM2 (sTREM2), which has recently been shown to bind AβOs and inhibit Aβ oligomerization and fibrillization, blocking Aβ-induced neurotoxicity. These effects are lessened with an AD-risk variant of sTREM2 [38,39]. TREM2 is suggested to play a protective role by enabling microglia to surround Aβ plaques and alter their structure, thereby limiting neuritic damage [40,41].

Taken together, these observations indicate that AβOs and microglia are closely associated with each other and that this association is profoundly involved in AD pathology. Accordingly, modulating AβO-induced microglial activation has become an emerging strategy in the development of AD therapeutics.

Besides neurotoxicity and neuroinflammation, Aβ42 or Aβ42 oligomers were reported to disrupt the BBB, which may be mediated by the upregulation of RAGE [42,43]. RAGE is known to mediate Aβinflux across the BBB and is also implicated in Aβ cytotoxicity [44].

## 3. Small Molecules with AβO Toxicity-Reducing Activity 

Among the small molecules capable of reducing AβO-mediated toxicity highlighted in my previous review were the natural compounds tyrosol, honokiol, and rhynchophylline (Rhy). Notably, almost all of these molecules have potent antioxidative activity, underscoring the central role of oxidative stress in the pathophysiological cascade of AβO toxicity, as described above [17]. In reviewing the signaling pathways involved in the AβO toxicity-reducing activity of these molecules, it became apparent that most of these molecules have the capacity to activate the Nrf2 pathway and initiate antioxidant defense responses. Nrf2 is a transcription factor that is well established as a key transcriptional regulator of cellular responses to oxidative stress [18]. Phosphoinositide 3-kinase (PI3K)/Akt and glycogen synthase kinase 3-beta (GSK3β) pathways appear to be relevant to the activation of Nrf2 by some of these molecules. In addition, some of these molecules are also able to activate the PPARγ pathway or modulate other pathways, such as SIRT3 (sirtuin 3), NF-κB (nuclear factor kappaB), and c-Jun N-terminal kinase 3 (JNK3)/p38 pathways [17].

PPARγ is a member of the PPAR family of ligand-activated nuclear receptors that acts as a transcription factor to regulate various functions, including mitochondrial function and antioxidant defense [19,20]. Importantly, Nrf2 and PPARγ are interrelated [45]. Specifically, an Nrf2 deficiency leads to decreased expression of PPARγ [46], and conversely, Nrf2 activation enhances PPARγ expression [47]. Thus, Nrf2 can regulate PPARγ. 

Notably, some small molecules can activate both Nrf2 and PPARγ pathways, a feature that likely underlies their significant protective action against AβO toxicity [17].

## 4. Nrf2, AβO Toxicity, and AD Pathology

The Nrf2 system is a fundamental defense system against oxidative stress that is regulated by both Keap1-dependent and Keap1-independent mechanisms [18,48]. In the Keap1-dependent mechanism, activation of Keap1, a cytoplasmic inhibitor of Nrf2, by certain stimuli causes Nrf2 release and translocation to the nucleus, where it binds to antioxidant response elements (ARE) to induce the expression of antioxidant and metabolic genes. In the Keap1-independent mechanism, Nrf2 is regulated by the signaling mediator, GSK3β. GSK3β can phosphorylate Nrf2, leading to the recognition of phospho-Nrf2 by an E3 ligase receptor and the F-box protein β-TrCP, followed by its proteosome-mediated degradation [49]. GSK3β also can phosphorylate Fyn, which in turn regulates Nrf2 via phosphorylation [50].

Intriguingly, Nrf2 appears to be dysregulated in the AD brain, as evidenced by a reduction in the levels of nuclear Nrf2 in cortical and hippocampal tissues of AD patients [51]. Another study by Bahn et al. found that Nrf2 expression is reduced in AD brain samples, which may be related to Aβ accumulation [52]. Furthermore, studies using animal models have suggested a direct association between Nrf2 and AD pathology. For example, AD model mice lacking Nrf2 show an increase in astrocytes and microglia and increased levels of interferon (IFN)-γ and exhibit worsened cognitive deficits [53,54]. Bahn et al. [52] showed that Nrf2 can also negatively regulate BACE1 expression through binding to ARE sites in the *BACE1* promoter. These researchers showed that an Nrf2 deficiency increases BACE1 expression and exacerbates Aβ plaque loads and cognitive deficits in 5XFAD mice.

Conversely, Uruno et al. [55] demonstrated that induction of Nrf2 in APP knock-in AD model mice through a genetic reduction in Keap1 suppresses oxidative stress and activation of microglia and astrocytes. These mice also show improvement in cognitive performance. These authors further found that intraperitoneal administration of the natural compound 6-MSITC, an Nrf2 inducer, ameliorated cognitive impairment in AD model mice. 

An interesting Nrf2-activating compound is carnosic acid (CA), a component of rosemary and sage. Lipton and coworkers showed that CA, an electrophilic drug, is activated by ROS [56]. Upon activation, it reacts with a thiol group on Keap1, resulting in Nrf2 activation. CA was further shown to reduce AβO-induced spine loss in primary cortical neurons, and when intranasally administered for 3 months, it rescued dendritic and synaptic loss, astrocytosis, and Aβ accumulation in hAPP-J20 mice. CA treatment also mitigated cognitive impairment in these mice [57]. Thus, CA appears to be a promising candidate molecule for counteracting AβO toxicity. 

Honokiol, a phenolic compound found in *Magnolia officinalis*, has been shown by several studies to prevent AβO toxicity through its antioxidative action, as summarized previously [58,59]. A recent study by Hou et al. [60] revealed that honokiol alleviates oxidative stress-induced neurotoxicity in PC12 cells through Nrf2 activation. They postulated that honokiol forms a quinone intermediate upon oxidation that is reactive and modifies sulfhydryl groups in Keap1, leading to the dissociation of Keap1 from Nrf2. 

Other small molecules that can reduce AβO toxicity and activate Nrf2 include Rhy, caffeic acid phenyl ester (CAPE), nicotinamide mononucleotide (NMN), tyrosol/hydroxytyrosol, and ferulic acid [17].

Jiang et al. [61] reported that Rhy, a biological component of *Uncaria rhynchophylla*, protects against AβO-induced toxicity in AβO-injection model mice through activation of Nrf2. Rhy administration was also shown to be capable of penetrating the BBB and ameliorating Aβ pathology and neuroinflammation in APP/PS1 mice [62]. The protective action of Rhy against AβOs may also be mediated by the antagonism of NMDA receptors containing GluN2B subunits [63].

CAPE, an active component of propolis, has a broad spectrum of pharmacological activities, including antioxidant and anti-inflammatory properties [64]. CAPE administration was shown to prevent oxidative stress and neuroinflammation and reverse cognitive impairment in AβO-injected mice—effects that appeared to be mediated by Nrf2 activation [65]. 

NMN is a precursor of NAD^+^ that has neuroprotective effects against various stimuli, including oxidative stress [66]. NMN prevents AβO-induced neuronal death and inhibition of long-term potentiation (LTP) in organotypic slices; it also decreases Aβ accumulation and inflammatory responses in AD model mice. In an intracerebral hemorrhage mouse model, NMN treatment was found to significantly reduce brain edema, brain cell death, oxidative stress, and neuroinflammation, all of which were apparently mediated by Nrf2 activation [67].

Tyrosol and hydroxytyrosol, antioxidative phenols found in olives [68], and ferulic acid, an antioxidant found in plant cell walls, exert protective effects against AβOs in vitro and in vivo [69,70,71,72,73] and also have been shown to enhance the Nrf2 pathway in other models [74,75,76].

Recent studies have reported a functional connection between Nrf2 and macroautophagy, demonstrating, for example, that Nrf2 levels are regulated by the autophagy-related adaptor protein p62 [77]. In this regard, it is of interest that Nrf2 activation may also reduce phosphorylated tau protein via the autophagy-lysosome pathway through the induction of the autophagy adaptor protein NDP52 [78]. Rojo et al. [79] reported that an Nrf2 deficiency increased insoluble mutant tau levels in double transgenic mice expressing APP (V717I) and tau (P301L). Further studies are required to elucidate whether Nrf2 activation can reduce phosphorylated tau in the brains of tauopathy mice. 

## 5. PPARγ, AβO Toxicity, and AD Pathology

As noted above, PPARγ acts as a transcription factor that regulates genes implicated in various biological processes, including survival, glucose metabolism, oxidative stress, and neuroinflammation. Accordingly, PPARγ confers protection under pathological conditions, as reviewed elsewhere [19,20]. PPARγ is expressed in both neurons and glial cells in the brain, and although PPARγ signaling targets multiple processes, its modulation of mitochondrial function and neuroinflammation is particularly important in relation to AD. In this context, PPARγ enhances the expression of PGC1-α (PPARγ coactivator), which plays important roles in mitochondrial biogenesis and cellular energy metabolism [20]. PGC1-α is expressed in the brain, and its expression is reported to be decreased in brain tissues of AD patients [80]. Consistent with this relationship, stimulation of PPARγ is reported to promote mitochondrial biogenesis [20,81]. It is also suggested that Nrf2 is controlled by PGC1-α [81]. 

A number of studies have also shown that PPARγ negatively modulates neuroinflammation [82]. It is of particular significance that PPARγ negatively regulates NF-κB activity through trans-repression mechanisms. One such mechanism that has been proposed is that PPARγ interacts with NFκB p65/p50 to repress its transcriptional activity [83]. PPARγ may also act as a ubiquitin ligase to promote the degradation of p65 [84]. 

Type 2 diabetes is a risk factor for AD, and systemic and brain insulin resistance appear to be linked. Because amyloidogenesis and insulin resistance are intimately associated with each other, brain insulin resistance is suggested to be critically involved in AD pathophysiology [29]. In this regard, PPARγ is an important factor that can possibly ameliorate the defective insulin signaling in AD.

A number of PPARγ activators have been reported to prevent AβO-induced toxicity and/or neuroinflammation in various models. Thiazolidinediones (TZDs), the most popular PPARγ agonists, are used clinically in the treatment of diabetes mellitus. TZDs such as pioglitazone (Pio) and rosiglitazone (Rosi) have been shown to have beneficial effects in various in vitro and in vivo AD models. In particular, some studies have found that TZDs can prevent Aβ toxicity, including AβO toxicity. For example, Inestrosa et al. [85] reported that Rosi or troglitazone prevented neuronal degeneration and increases in GSK3β activity and cytoplasmic Ca^2+^ induced by Aβ40. Xu et al. [86] showed that Rosi prevented AβO-induced synaptic disturbances in cultured hippocampal neurons and also attenuated AβO-induced LTP deficits in hippocampal slices. These researchers further suggested that the protective effects of Rosi are attributable to an increase in mitochondrial number. In another study by Xu et al. [87], Rosi was found to prevent memory deficits in mice induced by AβOs. Interestingly, they showed that Rosi inhibited microglia activation as well as increases in IL-1β and TNFα. In addition, Landreth and co-workers reported that Pio stimulated Aβ degradation by microglia and astrocytes and that treatment with Pio for only 9 days suppressed neuroinflammatory responses, enhanced microglial phagocytosis of Aβ, and reversed cognitive deficits in APP/PS1 mice [88]. Heneka and co-workers showed that Pio and DSP-8658, a PPARα/γ agonist, specifically enhanced Aβ phagocytosis in primary microglia, an effect that was mediated by upregulation of the pattern-recognition receptor, CD36. They also demonstrated that oral administration of DSP-8658 for 3 months not only induced Aβ phagocytosis and recruitment of microglia to Aβ plaques in APP/PS1 mice but also reduced Aβ burden and improved spatial memory performance in these mice [89]. 

Du et al. [90] showed that Rosi increased mRNA and protein levels of insulin-degrading enzyme (IDE), an Aβ-degrading protease, in neurons in a PPARγ-dependent manner. Further, PPARγ was shown to contribute to the upregulation of IDE by insulin receptor signaling. Quan et al. [91] also reported that Pio treatment of neurons treated with Aβ42 increased the expression of IDE mRNA and protein. Thus, it is likely that PPARγ activation can promote Aβ degradation through the transcriptional regulation of IDE.

Another recent study showed that treatment with a low dose of Pio for 7 weeks increased LRP1 expression and reduced Aβ40 levels in the hippocampus of SAMP8 mice [92]. LRP1 has a key role in clearing Aβ via transport across the BBB [93]. In this regard, it is noteworthy that low doses of Rosi upregulated mRNA and protein levels of LRP1 and increase Aβ uptake in endothelial cells [94]. Consistent with this, Wang et al. [95] observed that treatment with Rosi or Pio induced LRP1 expression and suppressed expression of RAGE in brain microvessels of ob/ob mice. It is also notable that Pio can activate Nrf2 in some neuronal models [96,97]. A number of clinical trials have tested TZDs, such as Pio and Rosi, for AD, but they failed to show clinical benefits [98].

In a further example, Wang et al. [99] reported that telmisartan, an angiotensin II receptor antagonist and PPARγ activator used as an antihypertensive medication, specifically inhibited neuroinflammation induced by AβO in microglial BV2 cells. This effect was likely mediated by PPARγ/PTEN pathways.

Curcumin, a natural constituent of turmeric, is known to exert neuroprotective effects in various models, including AD models, and to inhibit Aβ aggregation [100]. Liu et al. [101] used neuronal and glial mixed cultures and APP/PS1 transgenic mice to show that curcumin protected cholinergic neurons from Aβ toxicity and attenuated neuroinflammatory responses through NF-κB and PPARγ pathways. Zheng et al. [102] also showed that oral administration of curcumin reduced BACE1 levels and Aβdeposition and improved cognitive impairment in 5XFAD mice. Curcumin may also attenuate AβO toxicity through modulation of Aβ aggregation [103]. A recent clinical study indicated that oral ingestion of a bioavailable form of curcumin led to significant memory and attention benefits in non-demented adult subjects [104]. 

Small molecules that are capable of reducing AβO toxicity and activating both Nrf2 and PPARγ are exemplified by astragaloside IV (ASIV) and cyanidin 3-glucoside (C3G). ASIV was reported to act as a PPARγ agonist and to exert antioxidative and neuroprotective effects through Nrf2 activation [105,106]. Wang et al. [106] showed that ASIV prevented AβO-induced death of neuronal HT22 cells. They also demonstrated that oral administration of ASIV prevented neuronal loss and apoptosis and ameliorated cognitive impairment in a PPARγ-dependent manner in AβO-injected mice. In a similar mouse model, Chen et al. [107] showed that ASIV ameliorated microglial activation and cognitive impairment. ASIV was also shown to exert inhibitory effects on BACE1 expression, leading to reductions in Aβ levels and Aβ plaques in APP/PS1 mice [108]. Notable in this context, *BACE1* gene expression was previously shown to be negatively regulated by PPARγ activation [109]. 

Studies have also tested the efficacy of C3G, a dietary anthocyanin that has been reported to act as an antioxidant and anti-inflammatory agent, in mouse models of AD. Treatment with C3G was shown to protect SH-SY5Y cells from AβO or Aβ25–35 neurotoxicity in association with the upregulation of PPARγ [110]. Notably, oral administration of C3G alleviated cognitive deficits in APP/PS1 mice [111]. Sanjay et al. [112] reported that C3G upregulated PPARγ expression and reduced inflammatory cytokines and ROS, shifted the M1 phenotype of microglia to M2, and enhanced phagocytosis of Aβ42 in APP/PS1 mice. C3G and anthocyanins were also shown to activate Nrf2 in other models [113,114].

Honokiol has stimulatory effects not only on Nrf2 but also on PPARγ. Wang et al. found that treatment with honokiol downregulated BACE1 and reduced Aβ deposition in APP/PS1 mice; it also suppressed neuroinflammation and improved cognitive impairment in these mice. Importantly, these ameliorative effects were blocked by GW9662, a PPARγ antagonist [115].

## 6. Future Perspectives

It has been well established that AβOs are critically involved in the early pathogenesis of AD. However, unanswered questions concerning the Aβ oligomer hypothesis remain. For example, which receptors of AβOs are most critical? Which AβO species are most toxic? Additionally, how do AβOs affect tau or microglia? Despite these uncertainties, it is reasonable to target AβOs for the treatment and prevention of AD. In fact, a recently developed Aβ antibody (BAN2401) specific for Aβ protofibrils was reported to significantly delay the progression of cognitive impairment in early AD patients, including those with MCI due to AD [116,117]. This antibody exhibits a strong binding preference for Aβ protofibrils [116], which not only exert toxicity on neurons but also induce activation of microglia [118,119]. The success of BAN2401 thus appears to reflect its specificity for Aβ protofibrils. Smaller AβOs may also be important, and antibodies targeting them, such as ACU193 [120], remain to be tested in clinical trials. However, these antibodies have disadvantages, such as poor BBB penetration and liability for the development of vasogenic edema [121].

**Figure 1:**
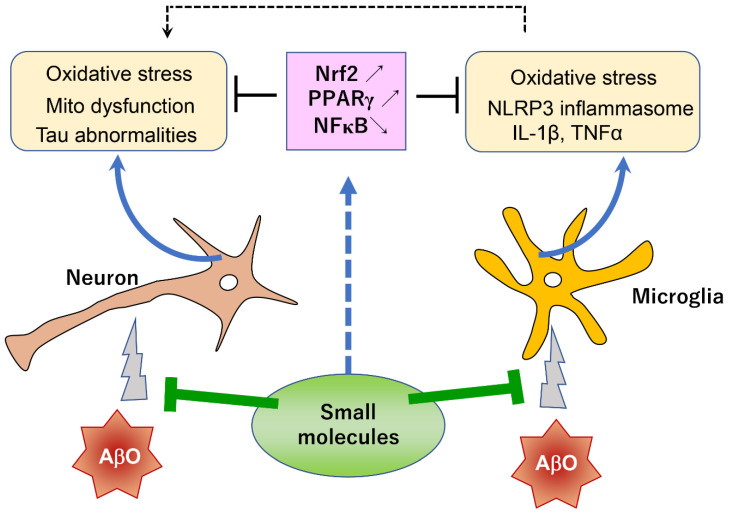
Important roles of Nrf2 and PPARγ pathways in the AβO toxicity-reducing effects of small molecules. AβOs act on both neurons and microglia, inducing neurodegeneration and neuroinflammation, respectively. Activated microglia produce various harmful factors that further aggravate neurodegeneration. AβO toxicity-reducing small molecules activate Nrf2 and/or PPARγ pathways, which can rescue both neurons and microglia through antioxidative and other mechanisms. These small molecules will be beneficial in preventing the pathological progression of AD. Mito dysfunction: Mitochondrial dysfunction.

In addition to such immunotherapeutic approaches, small molecule approaches for reducing the toxicity of AβOs are also therapeutically beneficial [17]. In the previous and present reviews of small molecules with AβO toxicity-reducing activity, I particularly emphasized the important roles of Nrf2 and PPARγ pathways in the mechanisms underlying the reduction in AβO toxicity. These Nrf2- and/or PPARγ-activating small molecules (Table 1) have several advantages. First, they are mostly of natural origin and can be used safely without serious side effects. Such a safety profile is highly advantageous in case they are used for a prophylactic purpose. Second, some of them can be administered orally and are capable of penetrating the BBB. Third, they can be used in combination with other drugs, including immunological agents, to produce a synergistic effect. Indeed, in light of the complex pathophysiology of AD, combination therapy is considered to be more feasible than monotherapy. Fourth, most of these small molecules have relatively simple chemical structures; thus, their pharmacological manufacture is likely straightforward. Finally, these molecules can act on both neuronal and glial cells to ameliorate neuronal dysfunction and neuroinflammation (Figure 1). Despite these advantages, clinical trials evaluating the efficacy of small molecules in reducing the toxicity of AβOs have been limited to TZDs. In their review of clinical trials of TZDs, Saunders et al. [98] noted that most of these studies were insufficiently powered or were not conducted long enough to detect changes with statistical confidence. Thus, the failure of TZDs in clinical trials is not a definitive indictment of their potential prophylactic effects.

## 7. Conclusions

I propose that AβO toxicity-reducing therapy (ATR-T) is a potentially beneficial strategy for the prevention of AD. Further preclinical and clinical studies are warranted to clarify whether ATR-T is effective in preventing the clinical progression from the MCI stage of AD to full manifestations of AD pathology. It will be essential to accurately diagnose patients with MCI due to AD using appropriate biomarkers and evaluate their clinical course for a sufficiently long period. Such clinical trials will hopefully verify the feasibility of the ATR-T concept in the prevention of AD.

## Figures and Tables

**Table 1:** Small molecules that reduce AβO toxicity and activate Nrf2 and/or PPARγ.

Compound	MW	Nrf2 Activation	PPARγ Activation	Refs	
				AD Models	Other Models
Carnosic acid	332	+		[57]	[56]
Honokiol	266	+	+	[58,59,115]	[60]
Astragaloside IV	785	+	+	[106,107,108]	[105]
C3G	450	+	+	[110,111,112]	[113]
Rhynchophilline	384	+		[61,62]	
CAPE	284	+		[65]	
NMN	334	+		[67]	
Tyrosol/H-Tyr	138/154	+		[69,70,71]	[74,75]
Ferulic acid	194	+		[72,73]	[76]
Pio/Rosi	356/357	+	+	[85,86,87,88,89]	[96,97]
Telmisartan	515		+	[99]	
Curcumin	368		+	[101,102,103]	

C3G: Cyanidin 3-glucoside; CAPE: Caffeic acid phenyl ester; H-Tyr: Hydroxytyrosol; NMN: Nicotinamide mononucleotide: MW: molecular weight; Pio: Pioglitazone; Rosi: Rosiglitazone.

## Data Availability

Not applicable.
